# Superoxide Dismutases in Pancreatic Cancer

**DOI:** 10.3390/antiox6030066

**Published:** 2017-08-19

**Authors:** Justin G. Wilkes, Matthew S. Alexander, Joseph J. Cullen

**Affiliations:** 1 Departments of Surgery and Radiation Oncology, University of Iowa Carver College of Medicine, Iowa City, IA 52245, USA; justin-wilkes@uiowa.edu (J.G.W.); matthew-alexander@uiowa.edu (M.S.A.); 2 Veterans Affairs Medical Center, Iowa City, IA 52245, USA

**Keywords:** pancreatic cancer, superoxide dismutase, NADPH oxidase, superoxide, manganese superoxide dismutase, extracellular superoxide dismutase

## Abstract

The incidence of pancreatic cancer is increasing as the population ages but treatment advancements continue to lag far behind. The majority of pancreatic cancer patients have a K-*ras* oncogene mutation causing a shift in the redox state of the cell, favoring malignant proliferation. This mutation is believed to lead to nicotinamide adenine dinucleotide phosphate (NADPH) oxidase activation and superoxide overproduction, generating tumorigenic behavior. Superoxide dismutases (SODs) have been studied for their ability to manage the oxidative state of the cell by dismuting superoxide and inhibiting signals for pancreatic cancer growth. In particular, manganese superoxide dismutase has clearly shown importance in cell cycle regulation and has been found to be abnormally low in pancreatic cancer cells as well as the surrounding stromal tissue. Likewise, extracellular superoxide dismutase expression seems to favor suppression of pancreatic cancer growth. With an increased understanding of the redox behavior of pancreatic cancer and key regulators, new treatments are being developed with specific targets in mind. This review summarizes what is known about superoxide dismutases in pancreatic cancer and the most current treatment strategies to be advanced from this knowledge.

## 1. Introduction

Adenocarcinoma of the pancreas is the fourth leading cause of cancer-related death in the United States [1]. Therapeutic responsiveness of pancreatic cancer to surgery, chemotherapy, and radiation therapy is poor, resulting in a dismal 5-year survival of less than 3% [2]. Mutation of the K-*ras* oncogene is a common event in the early development of pancreatic cancer occurring in 95% of pancreatic cancers, with a resulting overall increase in production of reactive oxygen species (ROS) [3]. Superoxide dismutases (SODs) modulate the oxidative status of the cell by dismutation of two molecules of O_2_^●−^ into hydrogen peroxide (H_2_O_2_) and molecular oxygen (O_2_) [4]. SODs have been shown to inhibit the in vitro and in vivo growth of pancreatic cancer through various intracellular signaling pathways [5,6]. Loss of MnSOD function leads to intracellular signaling supporting pancreatic acinar malignant cellular programming, while loss of extracellular superoxide dismutase (EcSOD) leads to an influx of regulatory pathways supporting pancreatic tumor microenvironments [7,8]. Therefore, we propose that strategies to scavenge mitochondrial and non-mitochondrial-generated O_2_^●−^ may prove beneficial in the treatment of pancreatic cancer. This review will focus on the role of SOD-induced inhibition of tumor growth and propagation, and its potential as a targeted pancreatic cancer therapy.

## 2. Pancreatic Adenocarcinoma

53,670 patients are projected to be diagnosed with pancreatic adenocarcinoma in 2017, making it the tenth most common cancer among both men and women; an incidence of 14.0 and 10.9 per 100,000, respectively [1,2]. Despite the relatively modest incidence, it is the fourth deadliest cancer amongst both men and women in the U.S. [1,2]. Over 43,000 patients are expected to succumb to the disease in 2017, with a death rate of 12.6 men and 9.6 women per 100,000. This high lethality is likely due to a combination of late stage at diagnosis, at which point 29% of patients will already have regional lymph node spread and 52% will have metastatic disease, and poor survival at all stages [1,2]. The relative lack of progress in therapy for pancreatic adenocarcinoma compared to other common cancers has led to opportunities for innovation and the development of new treatment modalities [9]. 

## 3. Superoxide and Superoxide Dismutase

**S**uperoxide (O_2_^●−^) is a byproduct of respiration and acts as a signaling molecule through one-electron oxidations (*via* HO_2_^•^) or reductions (*via* O_2_^●2−^), while hydrogen peroxide (H_2_O_2_), the byproduct of superoxide dismutation *via* SOD, participates in two-electron signaling circuits [10,11,12]. The aberrant passage of electrons to ~0.1–0.5% of oxygen molecules during respiration results in mitochondrial O_2_^●−^ generation [13]. In addition to the electron transport chain, other sources of O_2_^●−^ include NADPH oxidases (NOX) and xanthine oxidase [14]. The primary roles of NOX enzymes are to produce reactive oxygen species such as O_2_^●−^ [15]. For example, the immune system generates abundant O_2_^●−^ levels *via* NOX enzymes as an oxygen-dependent defense mechanism within phagocytes [12]. The NOX family consists of several homologs, which have the ability to deliver electrons across cellular membranes to generate these reactive oxygen species. NOX activation results in the regulation of gene expression, cell signaling, and cell differentiation, and is precisely why this family of enzymes has been linked to an equally wide range of pathologic states [15].

SODs are a class of metalloenzymes that scavenge O_2_^●−^ to prevent cell toxicity and regulate cell signaling. SODs are enzymes that participate in two-step reactions to dismute two molecules of O_2_^●−^ into one molecule of O_2_ and one molecule of H_2_O_2_, which undergo either subsequent dismutation by the catalase to H_2_O and O_2_ or are reduced to water by peroxidases [4]. An important feature of SOD enzymes is that they are highly compartmentalized. In mammalian species, three SOD isotypes have been identified: Copper Zinc SOD (CuZnSOD, SOD1), Manganese SOD (MnSOD, SOD2), and extracellular SOD (EcSOD, SOD3). 

CuZnSOD is present in the cytosol, nucleus, peroxisomes, and intermembrane space of the mitochondria of human cells [16]. Following the translation of the CuZnSOD protein (Molecular Weight 32 kDa), the stable homodimer is held together by hydrophobic contact [17]. It becomes activated by copper chaperone for superoxide dismutase (Ccs1) by introducing copper and creating an intramolecular disulfide bond for enzymatic action [18]. Only the copper subunit is enzymatically active; the zinc subunit purely structural. The enzymatically active copper reacts in a ping-pong mechanism where one superoxide molecule is reduced to hydrogen peroxide and a second superoxide is oxidized to molecular oxygen [19]. MnSOD is composed of four identical 22 kDa subunits and is essential for cell survival because it protects the mitochondria from reactive oxygen species in aerobic systems [20]. In mice, heterozygous MnSOD knock-out leads to a 50% decrease in enzyme activity and age-dependent oxidative DNA damage in the nucleus and mitochondria. Complete knock-out leads to severe cardiomyopathy, oxidative mitochondrial damage, and early postnatal death [21]. MnSOD also plays a crucial role in cellular biology beyond mitigating oxidative stress. It acts as a bifurcation point from the one-electron radicals (O_2_^●−^) and two-electron radicals (H_2_O_2_) which modulate biological stages of differentiation, proliferation, quiescence, apoptosis, and necrosis [22,23]. The delicate balance of O_2_^●−^ and H_2_O_2_, which MnSOD partially regulates, modulates the expression of critical transcription factors such as HIF-1α, AP-1, NF-κB, and p53 [24].

EcSOD is the only isoform of SOD that is expressed extracellularly and is a tetrameric glycoprotein containing four 30 kDa subunits, which utilizes copper and zinc ions in a similar fashion to the previously described soluble, cytosolic CuZnSOD (SOD1) [25,26,27]. EcSOD is cell type specific and is secreted in greatest amount by adipose tissue but also by the pancreas, lung, kidney, and vasculature [27,28]. It regulates the redox state of the extracellular environment and plays a key signaling role in the recruitment of inflammatory leukocytes and the creation of directly toxic molecules such as peroxynitrite [25]. Deficiency in mice results in increased oxygen-dependent toxicity [29]. A fundamental property of EcSOD, imparted by a heparin-binding domain (HBD) not found in other SODs, is its affinity for heparin sulfate proteoglycans located on the cell surface and in the extracellular matrix, allowing for its binding and adherence to the extracellular milieu [30]. Interestingly, the extracellular presence of superoxide dismutase has been observed to have anti-tumorigenic activity connected to intracellular signaling cascades and their downstream products. For example, the overexpression of EcSOD in vitro has been shown to inhibit the growth of melanoma by blunting tumor neovascularization through the down-regulation of vascular endothelial growth factor expression [31].

In general, cancer cells demonstrate an increase in the amount of reactive oxygen species present compared to normal tissues [32]. This is also true in pancreatic cancer as well [33]. Several studies have demonstrated high concentrations of reactive oxygen species are cytotoxic; however, at low concentrations, they regulate several key physiological processes including cell differentiation, apoptosis, and cell proliferation [32,34]. Redox-sensitive signal transduction pathways may control the regulation of these processes [35].

## 4. SOD, Superoxide and the Pancreas

The importance of functional SOD is demonstrated by its remarkable conservation. Population studies of functional polymorphisms have shown mixed results regarding risk for pancreatic cancer [36]. One small, case-control study demonstrated a two-fold increased risk for pancreatic cancer in patients with an alanine-to-valine polymorphism at codon 16 of the MnSOD gene, which is a part of the mitochondrial targeting sequence [37]. The limited evidence of genotypic variation is in spite of the very predictable pattern of SOD activity evolution with oncogenesis and suggests that these rare polymorphisms have a similar phenotype as the natural genotype, at least with regards to oncogenesis [38]. Regarding the systemic difference in SOD activity, some data identifies no difference in plasma SOD activity in patients with pancreatic cancer, suggesting any differences in activity driving tumorigenesis may be local phenomena [39].

**Figure 1:**
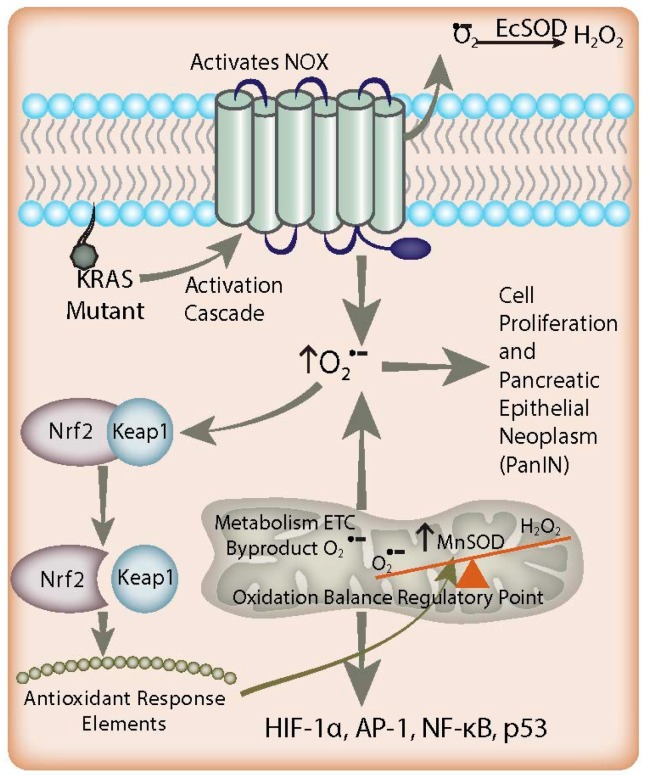
Redox-based mechanisms in pancreatic cancer. K-*ras* mutations and NADPH oxidase (NOX) overexpression lead to increased O_2_^●−^ flux in the early stages of pancreatic adenocarcinoma, resulting in activation of signaling pathways involved in proliferation. EcSOD scavenges the non-mitochondrial generation of O_2_^●−^, leading to growth inhibition in pancreatic cancer. In addition, there is an increased flux of mitochondrial O_2_^●−^ in combination with suppressed MnSOD expression found in pancreatic tumor development, resulting in a redox imbalance leading to cell signaling events contributing to proliferation. Simultaneously, increased O_2_^●−^ levels favor the dissociation of Nrf2 protein from Keap1. Nrf2 activates antioxidant response elements to increase the expression of MnSOD as a feedback mechanism to counterbalance the increased O_2_^●−^ levels generated by K-*ras* mutation via NOX. Overall, cell O_2_^●−^ levels remain increased and lead to PanIN malignant progression and Nrf2 expression; this paradoxically enables this progression by preventing cells from reaching cytotoxic O_2_^●−^ levels.

In pancreatic cancer, increased O_2_^●−^ acts as an antiapoptotic, pro-survival mediator (Figure 1) [40]. This is supported by increased levels of nitrotyrosine, a footprint of the reactive nitrogen species peroxynitrite (formed by the reaction of O_2_^●−^ with nitric oxide), in pancreatic cancer when compared to normal pancreas [41]. Growth factors were shown to stimulate O_2_^●−^ generation in pancreatic cancer cells through the activation of NOX, whereas inhibition of O_2_^●−^ production induced apoptosis. Similar results have been found in K-*ras* transformed human keratinocytes [42]. The increased levels of O_2_^●−^ could be reversed efficiently by intracellular SODs. Most interestingly, the results demonstrated that SOD promoted death in K-*ras* transformed cells, whereas non-K-*ras*-expressing or wild type K-*ras-*expressing cancer cells were unaffected [42]. This is important because 95% of pancreatic cancers are K-*ras* mutants. Mutation of K-*ras* alone is enough to induce tumorigenicity in previously non-tumorigenic, immortalized pancreas cells [43] Tumorigenesis from K-ras mutation follows a predictable progression from acinar cells to a progenitor ductal phenotype and, finally, to the early pancreatic intra-epithelial neoplasm (PanIN) [44,45,46]. The point mutation of the K-*ras* oncogene is a common event in the early development of pancreatic cancer leading to an overall increase in production of reactive oxygen species *via* activation of NOX [3]. Interestingly, fibroblasts transfected with the viral *ras* oncogene have a resulting increase in O_2_^●−^ production that has been proposed to act as a second messenger molecule to promote cell proliferation [47]. Oncogenesis mediated by *ras* also involves the activation of the Rel/nuclear factor (NF)-κB transcription factors. Indeed, constitutive activation of NF-κB p65 (RelA) has been demonstrated in 67% of pancreatic adenocarcinomas, which raises the possibility that it may itself be an oncogene playing a critical role in pancreatic tumorigenesis [48] Based on these observations, it is hypothesized that *ras* activates the NOX system to produce O_2_^●−^, which then leads to the activation of downstream signal transduction pathways, and ultimately cell proliferation.

K-*ras* mutation alone does not appear to decrease the SODs. Rather, upregulation of O_2_^●−^-producing NOX-2 and/or NOX-4 is likely an inciting initiating event which increases steady state reactive oxygen species in these cells (Figure 1) [33,44,49]. Furthermore, since K-*ras* is found in 95% of pancreatic cancers, this hypothesis could also explain the increased susceptibility of pancreatic cancer cells to scavenging of non-mitochondrial-generated O_2_^●−^ [5,6]. The mechanism by which K-*ras* is responsible for NOX-2/4 upregulation is still under investigation. Mitochondrial protein isolates from K-*ras* mutants demonstrate a decrease in the Complex I protein NADH dehydrogenase 1 alpha sub-complex assembly factor 1 (NDUFAF1) [50], with resultant depletion of the NAD^+^ pool. Recent data show that NOX4 may be able to use NADH as a substrate, which suggests that NOX4 upregulation is a necessary response to NAD^+^ pool depletion in K-*ras* mutants. There is also some evidence that inactivation of p16 (CDKN2A; a cyclin dependent kinase) is required for tumorigenesis and further NOX over-activity in vivo [44]. 

While SOD expression changes do not appear to be the inciting event in development, the subsequent down-regulation of SOD expression in early pancreatic cancer is well documented. In pancreatic cancer cells with low SOD expression, over-expression of all three types of SOD slow tumor growth, suggesting that tight regulation through the SODs is important for tumor survival [6]. The mechanism of slowed growth by SOD in pancreatic cancer is likely multifactorial. Clearly, certain signaling pathways are de-activated by O_2_^●−^ dismutation. The subsequent increases in H_2_O_2_ can be toxic, but smaller amounts can act as another growth signaling molecule, as demonstrated by additive decreased growth when SOD is combined with glutathione peroxidase (GPx) [51]. Thus, pancreatic cancer cells appear to be stimulated by increased levels of O_2_^●−^ and H_2_O_2_, which allows for growth stimulation without cytotoxicity [5,6,33]

One critical function of MnSOD is the redox-based regulation of the cell cycle, an oxidative signaling mechanism that appears to control cell progression through proliferative and quiescent phases. Cellular mitosis is driven by a highly choreographed series of fluctuations in the intracellular oxidative state [22,52,53]. There is an increase in MnSOD levels in the G_0_/G_1_ phase that fluctuates inversely with cellular superoxide levels, glycolysis, and oxygen consumption [54]. The “Warburg effect”, described nearly a century ago, is observed in cancer cells whereby mitochondrial damage leads to an increase in glycolysis [55]. There is now abundant data supporting the supposition that cancer cell mitochondrial damage is due to a loss in MnSOD expression leading to aberrant cell proliferation [7,56,57,58,59,60]. Indeed, MnSOD levels have been found to be lower in pancreatic cancer, which correlates with increased cell proliferative capability, an effect that is reversed when the cells are transfected to overexpress MnSOD [7]. 

The role of CuZnSOD in oncogenesis is poorly defined. Some, but not all, cancers demonstrate decreased amounts of CuZnSOD activity [61,62]. There is increased CuZnSOD activity in the hemolyzed erythrocytes of patients with pancreatic adenocarcinoma, although the clinical significance of this is unclear because, as stated earlier, total SOD activity does not appear to be affected [39,63]. MnSOD, however, appears to follow a very predictable pattern in pancreatic oncogenesis. The transition from an early pancreatic intra-epithelial neoplasm (PanIN) to advanced PanIN correlates strongly with loss of MnSOD expression (Figure 1) [64]. Gong, et al., demonstrated that the loss of Pigment Epithelial Derived Factor (PEDF) during this transition results in decreased NF-κB nuclear translocation and MnSOD promoter binding, resulting in decreased MnSOD transcription [64]. Concomitant is the loss of PEDF regulation of autophagy, which is also increased as early neoplasia matures [64]. Methylation of the MnSOD promoter has also been demonstrated to decrease MnSOD expression in pancreatic cancer cells [65].

In somewhat contradictory form, Pandit, et al., demonstrated increased NF-κB expression in pancreatic cancer via a positive feedback loop with miR301A [66]. While both studies demonstrate decreased MnSOD in pancreatic cancer, the difference may be in comparing PanIN and pancreatic adenocarcinoma where NF-κB seems to have opposite effects. Regardless, many studies have demonstrated increased early aggressiveness of pancreatic cancers expressing low levels of functional MnSOD, which is consistent with the low levels of MnSOD in primary tumors, and the reversal of this phenotype with MnSOD overexpression [7,67,68,69]. Pancreatic cancers also demonstrate lower MnSOD expression relative to normal tissue. Metastases have a trend toward elevated expression of MnSOD relative to primary tumors, but are still lower than the surrounding tissue into which they implant [70]. This adaptation may be necessary to survive epithelial-to-mesenchymal transition and a new tumor microenvironment. Experiments by Wenger, et al., in Syrian golden hamsters with pancreatic adenocarcinoma, induced via N-nitrosobis-2-oxopropylamine, demonstrated that liver metastases also have higher SOD activity than primary tumors, but consistently lower levels than surrounding liver tissue [71]. In contrast, Li et al., have demonstrated that more invasive pancreatic adenocarcinoma cells demonstrate higher levels of SOD^−^ and H_2_O_2_^−^ dependent NF-κB and ERK activation, which drives invasiveness [72,73]. The activated ERK pathway appears to activate invasive mediators like matrix metalloprotease 9 (MMP-9) and MMP-2 [73]. Their results may be explained by a few observations, starting with the fact that they were working with human specimens. Cells that survive chemo- or radio-therapy may develop resistance via up-regulation of MnSOD. Another confounding factor may be the heterogeneity of MnSOD expression within tumors. In fact, MnSOD appears to be regulated relative to the cell cycle, as there are specific pathways that will increase MnSOD in quiescent pancreatic cancer cells in a way that is necessary for their survival [74,75]. This makes a heterogeneous tumor environment much less predictable than cell culture, and renders some cancer cells less sensitive to chemo- and radiotherapy. 

Variant genotypes of EcSOD have been shown to be a risk factor for pancreatic adenocarcinoma [76]. Decreased EcSOD expression is associated with increased invasiveness secondary to increased O_2_^●−^ interaction with nitric oxide, while overexpression of EcSOD correlates with decreased HIF-1α and vascular endothelial growth factor (VEGF) expression, and decreased tumor growth [8,77]. These important data may implicate EcSOD as a critical regulator of redox conditions in the tumor microenvironment that facilitates tumor growth. 

The pancreatic tumor microenvironment (stroma) consists of fibroblasts, myofibroblasts, pancreatic stellate cells, immune cells, and endothelial cells [78]. Fibroblasts and extracellular matrix components are known to generate O_2_^●−^ in response to various cytokines to produce an ideal tumor microenvironment for supporting pancreatic cancer growth [79,80]. Cancer-associated fibroblasts (CAFs) have been identified as a key source of interleukin 6 (IL-6), which signals tumor inflammatory and immune responses, tumor proliferation, and tumor angiogenesis [81]. However, normal human fibroblasts (NHFs) have also been shown to exhibit increases in reactive oxygen species and mitochondrial abnormalities when chronically aged [82]. As a result, age-associated metabolic reprogramming may be occurring which could contribute to pancreatic cancer progression [82]. Interestingly, MnSOD activity can reverse increases in O_2_^●−^ generated by age-related mitochondrial morphology abnormalities. In fact, MnSOD has been shown in vitro to preserve mitochondrial morphology and protect fibroblasts from age-associated damage and chronological aging that can contribute to unregulated and aberrant proliferation [82].

## 5. Nrf2 Antioxidant Supports Pancreatic Cancer (PDAC) Development 

Nuclear factor erythroid 2-related factor 2 (Nrf2) is a transcription factor that allows healthy cells to maintain steady states in conditions of oxidative stress to prevent carcinogenesis [83]. In fact, Nrf2 is a master transcription factor thought to be responsible for inducing the expression of numerous antioxidant enzymes [84]. Normally, Nrf2 is bound to the Keap1 protein in the cytosol until oxidative stresses increase within the cell, causing Nrf2 to dissociate from the Keap1 protein [85]. Unbound Nrf2 is then able to translocate to the nucleus where it binds to antioxidant response elements (ARE), transcription factors for key antioxidant enzymes, including MnSOD [85,86]. While Nrf2 function suppresses cancer development in normal cells, abnormal constitutive activation in developing cancer cells can facilitate the protection and propagation of growing tumors [87]. In particular, Nrf2 activation was found to be a key regulator of tumor maintenance in pancreatic cancer [88]. This may seem contradictory to conventional wisdom, which suggests K-*ras* mutations lead to increased levels of ROS in acinar cells required for tumorigenesis [46]. However, while K-*ras* mutant cells do lead to downstream generation of ROS, and Nrf2 activity is increased in response [85], there is strong evidence supporting the presence of oxidative damage [45]. To reconcile these observations, it has been proposed that Nrf2 activity helps achieve a cellular redox balance allowing K-*ras*-induced oxidative stress to facilitate proliferation of PanIN lesions without becoming severe enough to induce senescence or cause toxic DNA damage [45]. Furthermore, Nrf2 activity may be an important source of chemoresistance in pancreatic tumor cells as a constant source of oxidative stress [89,90]. Targeting strategies to lower Nrf2 activity in tumor cells have been proposed that would theoretically eliminate the antioxidant counterbalance to K-*ras*-induced oxidative stress and remove this potentially important source of chemoresistance. Although there has yet to be full elucidation regarding Nrf2 regulation of any SOD isoform in pancreatic acinar cells, an important link does likely exist.

## 6. Redox-Based Therapy in Pancreatic Cancer

Treatments aimed at modulating oxidative stress in pancreatic adenocarcinoma have focused on both increasing and decreasing reactive oxygen species [91,92]. Drugs that increase O_2_^●−^ (Methyl-2-cyano-3,12-dioxooleana-1,9(11)-dien-28-oate [CDDO-Me], dicumarol) have been shown to downregulate important growth regulators in pancreatic cancer including hTERT, p-AKT, p-MTOR, NF-κB, c-myc, SP1, and telomerases, and to induce apoptosis [93,94]. Some of these drugs (capsaicin) have been shown to directly downregulate SOD activity, resulting in increased O_2_^●−^ levels, leading to mitochondrial damage and apoptosis, while other drugs disrupt the mitochondrial transport chain leading to increased O_2_^●−^ [95,96]. Drugs that decrease O_2_^●−^ levels appear to act as either scavengers themselves (Lipoxin) or as activations of pathways involved in scavenging (Octreotide, Vitamins A, C, and E) [67,73]. In one experimental animal model, vitamin supplementation decreased hepatic metastatic disease spread with concomitant increased SOD activity in the hepatic metastatic cells, as well as in normal surrounding hepatocytes [97]. This suggests that the oxidative state of potential metastatic sites is also an important factor. 

### 6.1. Pharmacologic Ascorbate

Pharmacologic ascorbate (high dose intravenous Vitamin C) has demonstrated an ability to generate selective cytotoxicity in pancreatic cancer cells when combined with radiation and chemotherapy by acting as a prodrug to increase the flux of H_2_O_2_ [98]. Recent studies have further elaborated on the mechanism supporting its selective cytotoxicity in cancer. Abnormal cancer cell mitochondrial metabolism leads to increased levels of O_2_^●−^ and H_2_O_2_, generating increased labile iron through disruptions in iron homeostasis [99]. Oxidation of pharmacologic ascorbate produces extra H_2_O_2_ and reacts with the excess pool of labile iron via Fenton chemistry to generate (OH) capable of producing oxidative DNA damage [100]. This hypothesis was supported by producing MnSOD knockout variants in A549 NSCLC cells, which are ordinarily relatively resistant to pharmacologic ascorbate. The MnSOD knockout cells were found to produce increases in O_2_^●−^ levels leading to increased labile iron stores and enhanced cytotoxic sensitivity to pharmacologic ascorbate. 

### 6.2. Manganoporphyrins

Manganoporphyrins (MnP) are a class of molecules originally developed as SOD mimetics with a central Mn(III) chelated in a porphyrin ring. This molecule behaves as an SOD mimetic (O_2_^●−^ dismutation) through a two-step cycling between Mn(III) and Mn(II). First, Mn(III) is reduced in the presence of O_2_^●−^ to Mn(II) and O_2_. Second, Mn(II) is oxidized by O_2_^●−^ to Mn(III) and H_2_O_2_ [100]. However, in the presence of pharmacologic ascorbate, MnPs act as superoxide reductases, not as dismutases. Mn(III) is reduced by pharmacologic ascorbate to Mn(II), which reacts with O_2_ to form O_2_^●−^, and subsequently H_2_O_2_ and O_2_ [100]. The reason for this altered chemistry is that the porphyrin complex affects the half-cell reduction potential of the Mn (III), a metal favoring reduction by pharmacologic ascorbate [100,101,102]. As a result, MnPs easily redox cycle with pharmacologic ascorbate to enhance oxidation and increase the flux of H_2_O_2_ [103]. These results were reproduced in vivo, demonstrating decreased tumor volumes when MnP was combined with pharmacologic ascorbate and gemcitabine in pancreatic cancer mouse xenografts [104]. These experiments also determined decreases in tumor growth to be H_2_O_2_ mediated [104]. A Phase I clinical trial was completed, demonstrating the safety and tolerability of pharmacologic ascorbate in combination with gemcitabine for advanced pancreatic cancer [105]. Clinical trials are also underway combining pharmacologic ascorbate with gemcitabine and radiation for advanced local disease. However, the use of MnPs for pancreatic cancer have yet to make it to clinical study. Despite of the original design of MnPs to behave as SOD mimetics, the mechanisms linked to cytotoxicity in pancreatic cancer were not proven to be strictly related to its ability to dismute O_2_^●−^, but rather by its ability to enhance ascorbate oxidation [103,104]. More data is needed regarding the safety of MnPs in humans before combination regimens with pharmacologic ascorbate can be explored, but this treatment strategy does show promise.

### 6.3. Induction of ROS

Drug therapies that work through the induction of reactive oxygen species (2-methoxyestradiol, radiation) increase the expression of MnSOD and contribute to their radio-chemotherapy resistance [106,107]. The increase in MnSOD is likely highly regulated and multi-factorial, but the radiation-induced activation of NF-κB, Nrf-2, and SIRT3, leading to increased MnSOD activity, has been described [108,109]. However, this upregulation may include more than a signal transduction. Cells treated with gemcitabine release exosomes that have relatively high levels of MnSOD and catalase mRNA transcripts. When other cells are pre-treated with these exosomes, they demonstrate increased resistance to gemcitabine, and decreased levels of reactive oxygen species, suggesting lateral transfer as a potential mechanism of resistance to chemotherapy [110]. K-*ras* mutant pancreatic cancer cells also exhibit intense macropinocytosis, which aids in the uptake of these exosomes, making this pathway a target of new therapies [111].

## 7. Conclusions

Alteration of the oxidative state of cells is tightly intertwined with genetic mutations and protein expression changes in the development of pancreatic adenocarcinoma. K-ras mutations and NOX overexpression lead to increased O_2_^●−^ flux early in pancreatic adenocarcinoma. These events lead to activation of the signaling pathways involved in proliferation. EcSOD or pharmacologically scavenging non-mitochondrial sources of O_2_^●−^ may represent new opportunities for therapeutic intervention in pancreatic cancer. Increased O_2_^●−^ flux combined with the suppressed MnSOD expression found in pancreatic tumor development also leaves cells vulnerable to therapies designed to take advantage of the imbalance in antioxidants and pro-oxidants. Treatments designed to overwhelm these vulnerable cells with an oxidative burst can activate pro-apoptotic pathways resulting in cell death. The resistance developed in surviving cells appears to be established by upregulation of the previously suppressed SODs.
